# Supplementary material for: Purification and characterization of antifungal lipopeptide produced by Bacillus velezensis isolated from raw honey
Source: PLoS One. 2022 Apr 6;17(4):e0266470. doi: 10.1371/journal.pone.0266470 (PMC8985968; doi:10.1371/journal.pone.0266470)

**S1 Fig.** LC-MS spectrum for singly charged m/z 1043.5 and doubly charged 522.3 of C_14_ iturin A. Spectrum was extracted from LC-MS for purified antifungal compounds produced by *Bacillus velezensis* WRB-ZX-001.


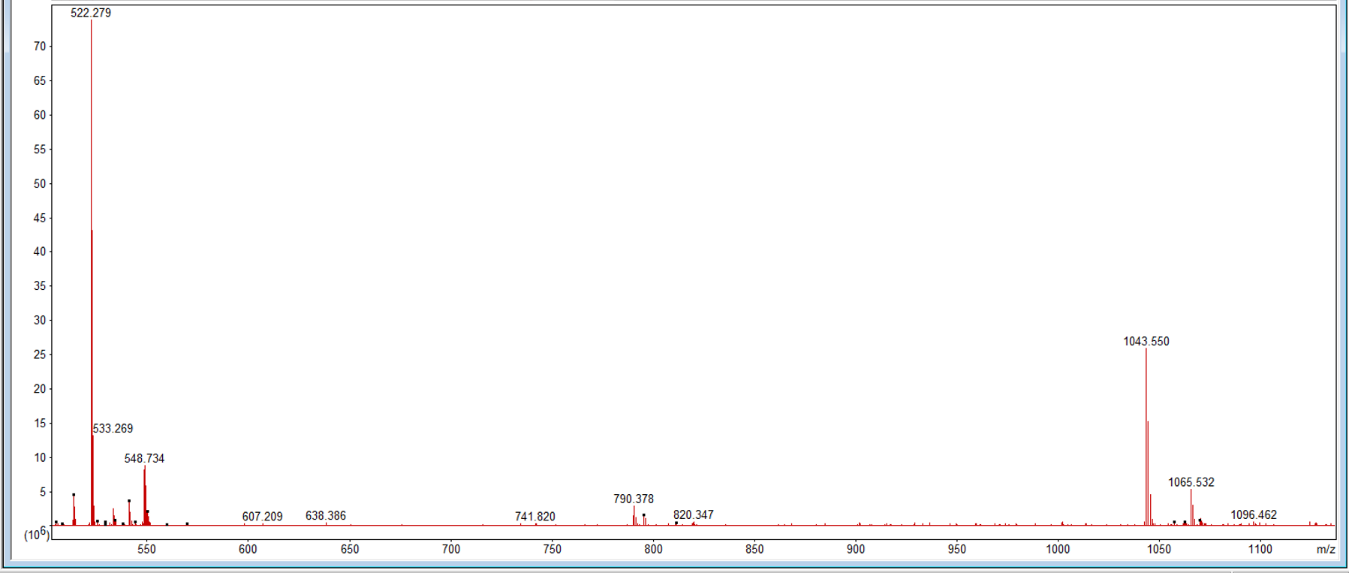

Supplement: S1 Fig — Spectrum was extracted from LC-MS for purified antifungal compounds produced by Bacillus velezensis WRB-ZX-001. (DOCX) [file pone.0266470.s001.docx]
