# Supplementary material for: Purification and characterization of antifungal lipopeptide produced by Bacillus velezensis isolated from raw honey
Source: PLoS One. 2022 Apr 6;17(4):e0266470. doi: 10.1371/journal.pone.0266470 (PMC8985968; doi:10.1371/journal.pone.0266470)
Supplement: S1 Table — (DOCX) [file pone.0266470.s002.docx]

**S1 Table.** Food-isolated fungal strains used in this study as indicators (adapted from Snyder, Churey, and Worobo (2019)).

| Organism | Strain ID | Food source |
| --- | --- | --- |
| *Syncephalastrum* | S11-0015 | Raw sprouted almonds |
| *Aspergillus* | S11-0016 | Nut mix |
| *Aspergillus* | S11-0033 | Oatmeal |
| *A. fumigatus* | S11-0039 | Kombucha |
| *A. niger* | S11-0054 | Pomegranate juice |
| *Rhodotorula* | S11-0057 | Red hot sauce |
| *P. glabrum* | S11-0071 | Hard-boiled egg |
| *Cladosporium* | S11-0111 | Juice beverage |
