# Supplementary material for: Purification and characterization of antifungal lipopeptide produced by Bacillus velezensis isolated from raw honey
Source: PLoS One. 2022 Apr 6;17(4):e0266470. doi: 10.1371/journal.pone.0266470 (PMC8985968; doi:10.1371/journal.pone.0266470)
Supplement: S2 Table — (DOCX) [file pone.0266470.s003.docx]

**S2 Table.** List of publicly available *Bacillus* spp. genome assembly included in this study.

| Isolate | Assembly Accession Number |
| --- | --- |
| B_amyloliquefaciens_ATCC_23350_strain_DSM7 | GCA_000196735.1 |
| B_amyloliquefaciens_strain_CC178 | GCA_000494835.1 |
| B_amyloliquefaciens_strain_DC-12 | GCA_000330805.1 |
| B_amyloliquefaciens_strain_EBL11 | GCA_000559145.1 |
| B_amyloliquefaciens_strain_EGD-AQ14 | GCA_000465655.1 |
| B_amyloliquefaciens_strain_HB-26 | GCA_000784675.1 |
| B_amyloliquefaciens_strain_IT-45 | GCA_000242855.2 |
| B_amyloliquefaciens_strain_KHG19 | GCA_000835145.1 |
| B_amyloliquefaciens_strain_LFB112 | GCA_000508265.1 |
| B_amyloliquefaciens_strain_LL3 | GCA_000204275.1 |
| B_amyloliquefaciens_strain_Lx-11 | GCA_001077735.1 |
| B_amyloliquefaciens_strain_TA208 | GCA_000195515.1 |
| B_amyloliquefaciens_strain_UASWS_BA1 | GCA_000469015.2 |
| B_amyloliquefaciens_strain_UMAF6614 | GCA_001593785.1 |
| B_amyloliquefaciens_strain_UMAF6639 | GCA_001593765.1 |
| B_amyloliquefaciens_strain_XH7 | GCA_000221645.1 |
| B_amyloliquefaciens_strain_Y2 | GCA_000262385.1 |
| B_siamensis_strain_7551 | GCA_002271775.1 |
| B_siamensis_strain_JJC33M | GCA_000798615.1 |
| B_siamensis_strain_KCTC_13613 | GCA_000262045.1 |
| B_siamensis_strain_SCSIO_05746 | GCA_002850535.1 |
| B_siamensis_strain_SRCM100169 | GCA_001662915.1 |
| B_siamensis_strain_XY18 | GCA_000966575.1 |
| B_subtilis_strain_168 | GCA_000009045.1 |
| B_velezensis_strain_AS43_3 | GCA_000319475.1 |
| B_velezensis_strain_CAU_B946 | GCA_000283695.1 |
| B_velezensis_strain_CBMB205 | GCA_002117165.1 |
| B_velezensis_strain_FZB42 | GCA_000015785.2 |
| B_velezensis_strain_KACC13105 | GCA_000960265.2 |
| B_velezensis_strain_KACC18228 | GCA_001461835.1 |
| B_velezensis_strain_KCTC13012 | GCA_001267695.1 |
| B_velezensis_strain_M27 | GCA_000299615.1 |
| B_velezensis_strain_NAU-B3 | GCA_000493375.1 |
| B_velezensis_strain_NJN-6 | GCA_000973585.1 |
| B_velezensis_strain_NRRL_B-41580 | GCA_001461825.1 |
| B_velezensis_strain_SK19_001 | GCA_000513755.1 |
| B_velezensis_strain_SQR9 | GCA_000685725.1 |
| B_velezensis_strain_TrigoCor1448 | GCA_000583065.1 |
| B_velezensis_strain_UCMB5033 | GCA_000455565.1 |
| B_velezensis_strain_UCMB5036 | GCA_000341875.1 |
| B_velezensis_strain_UCMB5113 | GCA_000455585.1 |
| B_velezensis_strain_YAU_B9601-Y2 | GCA_000284395.1 |
