# Supplementary material for: Purification and characterization of antifungal lipopeptide produced by Bacillus velezensis isolated from raw honey
Source: PLoS One. 2022 Apr 6;17(4):e0266470. doi: 10.1371/journal.pone.0266470 (PMC8985968; doi:10.1371/journal.pone.0266470)
Supplement: S3 Table — (DOCX) [file pone.0266470.s004.docx]

**S3 Table.** Gene Ontology (GO) annotation and node score distribution for *Bacillus velezensis* WRB-ZX-001 and WRB-ZX-002.

| **GO term** | *Bacillus velezensis* WRB-ZX-001 | *Bacillus velezensis* WRB-ZX-002 |
| --- | --- | --- |
| **Biological Process (BP) Number of Sequences** | 1953 | 1955 |
| Transport | 11% | 11% |
| Carbohydrate derivative metabolic process | 10% | 10% |
| Regulation of gene expression | 9% | 9% |
| Transcription, DNA-templated | 9% | 9% |
| Carbohydrate metabolic process | 8% | 8% |
| Alpha-amino acid metabolic process | 8% | 8% |
| Organonitrogen compound biosynthetic process | 7% | 7% |
| Nucleobase-containing small molecule metabolic process | 5% | 5% |
| Phosphate-containing compound metabolic process | 5% | 5% |
| Organophosphate metabolic process | 4% | 4% |
| Monocarboxylic acid metabolic process | 4% | 4% |
| Regulation of cellular macromolecule biosynthetic process | 4% | 4% |
| **Cellular Component (CC) Number of Sequences** | 1870 | 1870 |
| Integral component of membrane | 55% | 55% |
| Cytoplasm | 26% | 26% |
| Plasma membrane | 16% | 16% |
| Catalytic complex | 3% | 3% |
| **Molecular Function (MF) Number of Sequences** | 2660 | 2662 |
| Hydrolase activity | 32% | 32% |
| Oxidoreductase activity | 17% | 17% |
| Metal ion binding | 13% | 13% |
| Transmembrane transporter activity | 11% | 11% |
| DNA binding | 10% | 10% |
| ATP binding | 9% | 9% |
| Transferase activity; transferring phosphorus-containing groups | 8% | 8% |
