# Supplementary material for: Purification and characterization of antifungal lipopeptide produced by Bacillus velezensis isolated from raw honey
Source: PLoS One. 2022 Apr 6;17(4):e0266470. doi: 10.1371/journal.pone.0266470 (PMC8985968; doi:10.1371/journal.pone.0266470)
Supplement: Attachment — Submitted filename: Response to reviewers.docx [file pone.0266470.s006.docx]

We thank the editor for the opportunity to submit a revised version of our manuscript “Purification and characterization of antifungal lipopeptide produced by *Bacillus velezensis* isolated from raw honey”. We appreciate the feedback from the reviewers, and we have revised our manuscript based on the reviewers’ suggestions. We address the specific issues in a detailed response below.

***Reviewer #1: Introduction***

***- Line 45: In the medical field, ……… and safe to use. Is not clear, please rewrite this sentence***

We have re-written the sentence for better clarity.

***- Line 53 in the introduction, delete the dot after infection.***

We have made the suggested change.

***- Line 86: replace antioxidant by compounds***

We have made the suggested revision.

***- Line 89: the sentence “B. subtilis H215 was isolated from raw honey that was inhibitory to Byssochlamys fulva H25” need to be revised.***

We have revised the sentence for better clarity.

***- Line 91: the ref “Lee, Churey (16)” must be corrected as the journal instructions.***

We appreciate the reviewer’s comment and have corrected the reference as required by the journal.

***- Line 99: please change The potential application of these natural products from microbial source by The potential application of these microbial natural products***

We have made the suggested change.

***Results***

***- 121: The first sentence should be revised***

We have re-written the sentence for better clarity.

***- Line 123: please change this sentence “These 12 isolates were identified by 16S rRNA sequencing; all isolates were Bacillus spp.”*** ***by The 16S rDNA sequence of these 12 isolated strains showed a highest identity to that of several B. subtilis spp.***

We have re-written the sentence as suggested for better clarity.

***- Line 124: change the sentence “by ……were summarized respectively” in Table 1 and 2.***

We have re-written the sentence to avoid confusion.

***- Table 1: this table should be placed in material and methods***

We have rearranged the table order and put the original table 1 in the supporting information.

***- As described in table 2, I don’t understand why you have chosen only Co29 and Co30? and not isolates from clove honey like Co5 and Co-6?. These later have better antifungal activity.***

We appreciate the reviewer’s comment. As we mentioned in the manuscript (Line 130-132), the isolates Co29 and Co30 were the only two isolates that were able to produce and excrete antifungal compounds to the extracellular environment. Consequently, these two isolates were selected for the following experiments.

***- Legend of table 2 should be improved***

We have re-written the legends for better clarity.

***- m/z in italic***

We have made the suggested change.

***-line 165: “Based on results from previous studies, we presumed that the ions with m/z value of 1043.55 and 1057.57 were C14 and C15 iturin A [M+H]+, respectively: please add references and the formula of the identified compound. Indeed, precise that C14 was at ion with m/z value of 1057.57 was assigned to C15 iturin A [M+H]+. Ion with m/z value of 1079.55 was assigned to C15 iturin A [M+Na].***

We appreciate the reviewer’s comment and have added two references on Line 164-165. The molecular formula of the identified compound is also included on Line 165-166.

***- C14 or C15? Please clarify***

We thank the reviewer’s comment, but we think our phrasing is clear. The peak with m/z value of 1043.55 was C_14_ iturin A [M+H]^+^; m/z 1057.57 was C_15_ iturin A [M+H]^+^; m/z 1079.55 was C_15_ iturin A [M+Na]^+^ (Line 165-166, Fig 3 legend, S1 Fig legend).

***-Last paragraph page 11: add the figure number***

We appreciate the reviewer’s comment, but the paragraph mentioned by the reviewer was a summary of the genome sequencing data and there were no associated figures.

***-Figure legend (line 182): “was” and not “were”***

We have made the suggested change.

***-Line 209 to 210: sentence should be revised***

We have re-written the sentence for better clarity.

***-line 401-402: sentence should be revised***

We have revised the sentence for better clarity.

***Materials and methods***

***- Please add a title “Antifungal assays” before the paragraph in line 448***

We have made the suggested change.

***Additionally, the authors should check their English writing. It should be improved.***

We appreciate the reviewer’s comment. We have read through our manuscript and corrected any grammar or language issues.
